# A short period of anticoagulant treatment for venous thrombosis associated with osteomyelitis is feasible: 6 cases report

**DOI:** 10.1097/MD.0000000000042930

**Published:** 2025-06-13

**Authors:** Haiting Jia, Tao Liu

**Affiliations:** a Department of Orthopaedic Trauma Surgery, Children’s Hospital Affiliated to Shandong University (Jinan Children’s Hospital), Jinan, Shandong, China.

**Keywords:** case report, child, osteomyelitis, venous thrombosis

## Abstract

**Rationale:**

Venous thrombosis associated with osteomyelitis is rare in children, and there is no consensus on the duration of anticoagulation for thrombosis. The available literature reports long anticoagulation times for venous thrombosis.

**Patient concerns:**

The clinical data of 6 children with osteomyelitis with venous thrombosis admitted from 2018 to 2022 were retrospectively analyzed.

**Diagnoses:**

Osteomyelitis was diagnosed by magnetic resonance imaging, and venous thrombosis was detected by ultrasound.

**Interventions:**

Osteomyelitis was treated with antibiotics combined with surgical drainage. One child with venous thrombosis was not anticoagulated, and the remaining 5 children were on anticoagulant therapy for a maximum of 10 days.

**Outcomes:**

All 6 cases of osteomyelitis were cured, and the thrombus disappeared by regular ultrasound review.

**Lessons:**

From our experience in treating venous thrombosis in 6 children with osteomyelitis we believe that venous thrombosis associated with osteomyelitis does not require prolonged anticoagulation.

## 1. Introduction

Osteomyelitis refers to the infection and destruction of bone, and the causative organism is mostly *Staphylococcus aureus*. Osteomyelitis is usually a hematogenous infection and is one of the most common infectious diseases in children. Osteomyelitis, if not diagnosed and treated promptly, can lead to many complications such as limb deformity,athologic fracture, deep vein thrombosis(DVT), and even death.

Venous thrombosis is a rare complication of osteomyelitis. It has been reported that the prevalence of DVT among acute hematogenous osteomyelitis is 6% to 10%.^[1]^ There’s a link between thrombosis and *S aureus*. *S aureus* causes platelet aggregation as well as vascular endothelial cell damage by releasing exotoxins. In addition, the coagulase produced by S. aureus is able to activate the conversion of fibrinogen to fibrin, leading to blood clotting.^[2]^

The treatment of osteomyelitis is relatively well defined clinically, with treatments including antibiotics against infection and surgical drainage. In children, anticoagulation is the usual treatment for thrombosis. However, the duration of anticoagulation is still not standardized. Continued use of low molecular weight heparin until the DVT disappears has been reported in the literature.^[3]^ Does the treatment of venous thrombosis really require prolonged anticoagulation? We treated 6 cases of venous thrombosis associated with osteomyelitis, using heparin anticoagulation for a maximum of 10 days, and after at least 1 year of follow-up, all children had a favorable prognosis, with disappearance of the thrombus. We present this case series in accordance with the AME case series reporting checklist.

## 2. Cases report

The clinical data of 6 children with osteomyelitis with venous thrombosis admitted from 2018 to 2022 were retrospectively analyzed. The 6 cases included 5 males and 1 female aged from 4 months to 11 years, all presenting with localized limb swelling and pain. The osteomyelitis primarily involved the tibia (3 cases), femur (2 cases), and talus (1 case). Pathogens included methicillin-sensitive *S aureus* (2 cases), methicillin-resistant *S aureus* (3 cases), and 1 case with negative culture result. Osteomyelitis was treated with antibiotics combined with surgical drainage. Five patients received short-term anticoagulation therapy (heparin sodium/low molecular weight heparin) for 7 to 10 days, while 1 patient did not undergo anticoagulation, with the thrombus resolving spontaneously. Follow-up ultrasound examinations showed that all thrombi had disappeared, and complete vessel recanalization was observed within 6 months in all cases, with no persistent thrombosis or anticoagulation-related complications. The findings suggest that short-term anticoagulation therapy may be sufficient for venous thrombosis associated with osteomyelitis in children, with favorable outcomes. The clinical characteristics of 6 cases see, Table 1.

**Table 1:** Data of 6 children with osteomyelitis with venous thrombosis.

Case	Age	Duration of symptoms	Pathogen	D-dimer (reference value 0–1 mg/L)	Osteomyelitis site	Thrombosis site	Osteomyelitis treatment	Thrombosis treatment	Thrombus resolution time
1	1 yr	10 d	Negative	3.67 mg/L	Right talus	Right saphenous vein (calf)	Cefazolin + surgical drainage	No anticoagulation	2 wk
2	5 yr	4 d	MSSA	5.31 mg/L	Right tibia	Right saphenous vein (thigh)	Cefazolin + surgical drainage	Heparin sodium (10 d)	2 mo
3	4 yr	3 d	MRSA	3.86 mg/L	Left femur	Left femoral to popliteal vein	Vancomycin + surgical drainage	LMWH (6 d)	6 mo
4	10 yr	9 d	MRSA	2.42 mg/L	Right tibia	Right saphenous vein (calf)	Vancomycin + surgical drainage	LMWH (9 d)	1 mo
5	11 yr	7 d	MRSA	6.54 mg/L	Right femur	Right femoral to popliteal vein	Vancomycin + surgical drainage	LMWH (10 d)	6 mo
6	4 mo	4 d	MSSA	4.54 mg/L	Right tibia	Right ankle superficial vein	Cefotaxime + surgical drainage	Heparin sodium (7 d)	7 d

LMWH = low molecular weight heparin, MRSA = methicillin-resistant *Staphylococcus aureus*, MSSA = methicillin-sensitive *Staphylococcus aureus*.

## 3. Discussion

Thrombosis is usually secondary to surgery, indwelling vascular catheters, and less commonly to osteomyelitis. The 6 cases we reported were all related to osteomyelitis and not to indwelling vascular catheters.

D-dimer is a product of fibrin degradation, and elevated D-dimer indicates that the human organism is in a hypercoagulable state. All 6 children we reported had elevated D-dimer. We treated 94 cases of acute osteomyelitis from November 2017 to April 2022, of which 69 were *S aureus* infections and 25 had negative bacterial cultures. Elevated D-dimer was seen in 43 of 69 cases of acute osteomyelitis caused by *S aureus* infection and in 10 of 25 cases of acute osteomyelitis in which the causative organism was not cultured. There is no indication for routine prophylaxis of DVT to date.^[4]^ We also did not take anticoagulation prophylaxis for children with osteomyelitis with elevated D-dimer. Venous thrombosis in children is usually treated with anticoagulant drugs and rarely with surgery. The main anticoagulant drugs are heparin and warfarin. However, there is no consensus on the duration of anticoagulation. We reviewed relevant case reports via pubmed, and anticoagulation times of <10 days were rare.^[5]^ More cases reported anticoagulation for up to 6 months.^[6–8]^ It should be noted that prolonged anticoagulant drugs may affect the coagulation function of the child, and regular monitoring of coagulation function is required during use.

In 1 case, anticoagulant drugs were not used after the detection of thrombus, which was the 1st case of osteomyelitis with thrombus that we encountered, and we neglected to treat the thrombus due to our lack of knowledge about thrombus, but the thrombus disappeared on its own after 2 weeks. We used heparin in the remaining 5 children with osteomyelitis, none of them for more than 10 days. During our follow-up the venous thrombus disappeared and the vessels were recanalized in all of them. For this reason we propose whether venous thrombosis associated with osteomyelitis can be anticoagulated only in the acute phase of thrombosis. Due to our small number of cases, this needs to be verified with more cases.

## 4. Conclusions

We reported 6 cases of children with osteomyelitis with venous thrombosis, none of the venous thrombosis anticoagulation time exceeded 10 days, and all of the venous thrombosis disappeared through our follow-up, so we believe that a short period of anticoagulation for venous thrombosis related to osteomyelitis is feasible.

## Author contributions

**Conceptualization:** Haiting Jia.

**Data curation:** Haiting Jia.

**Formal analysis:** Haiting Jia.

**Writing – original draft:** Haiting Jia.

**Writing – review & editing:** Tao Liu.
